# High specificity of metagenomic next-generation sequencing using protected bronchial brushing sample in diagnosing pneumonia in children

**DOI:** 10.3389/fcimb.2023.1165432

**Published:** 2023-08-11

**Authors:** Chunyan Zhang, Zheng Li, Mengyuan Wang, Jiemin Zhou, Wenwen Yu, Huifang Liu, Bingxue Hu, Shifu Wang

**Affiliations:** ^1^ Department of Microbiology Laboratory, Children’s Hospital Affiliated to Shandong University, Jinan, China; ^2^ Department of Clinical Microbiology, Shandong Provincial Clinical Research Center for Children’s Health and Disease, Jinan, China; ^3^ Department of Scientific Affairs, Vision Medicals Center for Infectious Diseases, Guangzhou, China

**Keywords:** protected bronchial brush, MNGs, specificity, children, *Mycoplasma pneumoniae*

## Abstract

**Background:**

Lower respiratory tract infections are the leading cause of morbidity and mortality in children worldwide. Timely and accurate pathogen detection is crucial for proper clinical diagnosis and therapeutic strategies. The low detection efficiency of conventional methods and low specificity using respiratory samples seriously hindered the accurate detection of pathogens.

**Methods:**

In this study, we retrospectively enrolled 1,032 children to evaluate the performance of metagenomics next-generation sequencing (mNGS) using bronchoalveolar lavage fluid (BALF) sample and protected bronchial brushing (BB) sample in diagnosing pneumonia in children. In addition, conventional tests (CTs) were also performed.

**Results:**

The specificity of BB mNGS [67.3% (95% CI 58.6%–75.9%)] was significantly higher than that of BALF mNGS [38.5% (95% CI 12.0%–64.9%)]. The total coincidence rate of BB mNGS [77.6% (95% CI 74.8%–80.5%)] was slightly higher than that of BALF mNGS [76.5% (95% CI 68.8%–84.1%)] and CTs [38.5% (95% CI 35.2%–41.9%)]. During the epidemics of *Mycoplasma pneumoniae*, the detection rate of *M. pneumoniae* in the >6-year group (81.8%) was higher than that in the 3–6-year (78.9%) and <3-year groups (21.5%). The highest detection rates of bacteria, fungi, and viruses were found in the <3-year, >6-year, and 3–6-year groups, respectively. mNGS detection should be performed at the duration of 5–7 days after the start of continuous anti-microbial therapy or at the duration of 6–9 days from onset to mNGS test.

**Conclusions:**

This is the first report to evaluate the performance of BB mNGS in diagnosing pulmonary infections in children on a large scale. Based on our findings, extensive application of BB mNGS could be expected.

## Introduction

Lower respiratory tract infections are the leading cause of morbidity and mortality in children worldwide (Kyu et al., 2016; World Health Organization, 2018; Gabruseva et al., 2020), accounting for approximately 14% of all deaths in children aged <5 years in 2019 (Perin et al., 2022). Various pathogens can cause such infections (Kradin and Digumarthy, 2017; Lin et al., 2021), with the presenting symptoms of fever, cough, sputum production, dyspnea, pleuritic chest pain, and so on (Ruiz et al., 2000). Most importantly, *Mycoplasma pneumoniae* pneumonia accounts for 20%–40% of community-acquired bacterial pneumonia during epidemics (Waites et al., 2017). Due to the similar clinical manifestations among patients infected by different kinds of pathogens, accurate and timely etiological diagnoses are always difficult for clinicians (Cunha, 2006; Sheu et al., 2010). Meanwhile, some children may develop systemic complications, including sepsis and septic shock, metastatic infection, multiorgan failure, acute respiratory distress syndrome, or death, which prolong hospitalization, increase economic burden, and result in worse prognoses (de Benedictis et al., 2020). Accordingly, timely and accurate identification of pathogens and appropriate therapies is needed.

For infection diagnosis, bronchoalveolar lavage fluid (BALF) is an ideal sample (Langelier et al., 2018; Chen et al., 2020), while bronchial brushing (BB) sample is also used (Wang et al., 2020). Conventional tests (CTs) for diagnoses of pulmonary infections, including time-consuming and low-sensitivity culture methods, polymerase chain reaction (PCR), and serology tests with interpretational difficulties, are not satisfactory (Carroll and Adams, 2016; Buchan et al., 2021). More than 60% of pathogens cannot be detected by BALF culture (Chen et al., 2020), and a limited number of pathogens could be detected in a single experiment using hypothesis-based PCR (Marcilla-Vazquez et al., 2018) or antibody methods (Ge et al., 2021). In addition, limited volumes of samples obtained from children (Yagupsky and Nolte, 1990) further hindered the extensive application of CTs in accurate and rapid diagnoses. With the first successful application of metagenomics next-generation sequencing (mNGS) in clinical infection of the central nervous system (Wilson et al., 2014), this unbiased technology has been extensively used to detect pathogens from various infections (Wu et al., 2020; Chen et al., 2021a; Gu et al., 2021a), including pulmonary infection (Chen et al., 2020; Gu et al., 2021b).

Compared with CTs, unbiased mNGS can detect more microbes and trace pathogens from the samples, exhibiting significant advantages (Chen et al., 2021b; Chen H. et al., 2021). Chen et al. found that the sensitivity of BALF mNGS can reach up to 88.9%, while the specificity was only 14.9% (Chen et al., 2021b). This may be because some microbes not derived from pulmonary focal sites were included during the BALF sampling process, producing more false-positive results and decreasing the specificity of mNGS. A protected BB sampling process can realize relatively accurate microbial collection from pulmonary focal sites (Wang et al., 2020). Moreover, compared with BALF samples, BB samples were much more suitable for diagnosing pulmonary infections because the BB sampling process can reduce contamination, decrease the heart and lung load of children, and improve the positive rate of detection. Accordingly, we hypothesized that the specificity of BB mNGS was higher than that of BALF mNGS. However, few studies have been performed to evaluate the diagnostic values of mNGS using BB and BALF samples on a large scale.

To verify our hypothesis, we collected BALF and BB samples from children with suspected pneumonia during epidemics of *M. pneumoniae*. We performed both mNGS and CTs to test their accuracies against final clinical diagnoses. We also constructed a fitting model to evaluate the effects of empirical therapy on pathogen detection of mNGS and conventional methods and to identify the best timing for the mNGS test. In addition, we also summarized changes in medication according to the mNGS results.

## Methods

### Ethics statement

This study was reviewed and approved by the Ethical Review Committee of Children’s Hospital Affiliated with Shandong University (approval no. SDFE-IRB/P-2022017). All procedures followed were in strict compliance with the Ethical Review of Biomedical Research Involving Human Subjects (2016), the Declaration of Helsinki, and the International Ethical Guidelines for Biomedical Research Involving Human Subjects.

### Study design

From January 1, 2020, to April 30, 2022, 1,032 out of 1,129 children with pneumonia were enrolled in this retrospective study. The inclusion criteria were as follows: 1) new-onset radiological findings on chest X-ray or computed tomography and 2) at least one of the following typical clinical characteristics: a) new-onset cough, sputum production, dyspnea, chest pain, or exacerbation of existing respiratory symptoms; b) fever; c) clinical signs of lung consolidation or moist rales; and d) peripheral leukocytosis (>10 × 10^9^/L) or leucopenia (<4 × 10^9^/L). The exclusion criteria included the following: 1) the parents refused to sign informed consent, 2) mNGS was not performed, and 3) the patients had incomplete clinical information. Physical information and clinical characterization were collected. For children with pneumonia, BALF or BB samples were collected according to the judgment of the respiratory specialist.

For BB sampling, the bronchoscope with a protective sleeve at the end was first delivered to the lesion site. Brush reached the end of the bronchoscope through the bronchoscope working hole. Then, the brush was pushed out to let it reach the lesion site. Brushing tests were performed two to three times. After sampling, the brush was retracted into the protective sleeve and quickly removed from the bronchoscope. The brush was cut off using sterile scissors and put into 1 mL of sterile physiological saline, which was thoroughly mixed and used for mNGS and CTs. After signed informed consent was given, BALF or BB samples were collected. CTs included routine bacterial and fungal smears and cultures, India ink, acid-fast staining, serum antigen and antibody tests, interferon-γ release assays, tuberculin test, and PCR.

### Specimen processing and sequencing

BALF and BB samples were collected into sealed sterile tubes and temporarily stored below −20°C or transported to the laboratory on dry ice. According to the manufacturer’s instruction, 0.6 mL of BALF or BB samples with 250 mg of glass bead (0.5 mm) in a 1.5-mL microcentrifuge tube was first homogenized on a vortex mixer at 3,000 rpm for 30 min. For host cell depletion, 1 U of Benzonase (Sigma) and 0.5% Tween 20 (Sigma) were used. Then, 7.2 mL of lysozyme was added for a wall-breaking reaction. Approximately 0.3 mL of supernatant sample was separated into a new 1.5-mL microcentrifuge tube, and DNA was extracted using the TIANamp Micro DNA Kit (DP316, Tiangen Biotech, Beijing, China). DNA libraries were constructed through DNA fragmentation, end-repair, adapter ligation, and PCR amplification. Qubit 4.0 (Thermo Fisher Scientific, Waltham, MA, USA) was used to measure extracted DNA concentrations. Agilent 2100 (Agilent Technologies, Palo Alto, CA, USA) was used for quality control of the DNA libraries (Ji et al., 2020). Qualified libraries were pooled. DNA Nanoball was made and sequenced on MGISEQ-50, 200, or 2000 platform. On average, approximately 20 million single-end 50 base pair (bp) reads were obtained for each sample.

### Bioinformatics analysis

The obtained sequencing raw data were filtered to remove adapter and low-quality, low-complexity, and shorter reads (<35 bp). Human reads were removed by mapping to the human reference genome (hg38) using bowtie2, and the remaining sequences were aligned with the microbial genome database constructed according to the published microbial genome databases, including the reference sequence database at National Center for Biotechnology Information using Burrows-Wheeler Alignment (Li and Durbin, 2009).

As a control, negative and positive controls were also set with the same procedure and bioinformatics analysis. The reads per million (RPM) of each detected microorganism were calculated. For the detected bacteria, fungi, or parasites, a positive mNGS result was defined when the microorganism was not detected in the negative control (“No template” control (NTC)), and genome coverage of detected sequences belonged to this microorganism ranked top 10 among the microbes in the same genus or when its ratio of RPM_sample_ to RPM_NTC_ was >10 if the RPM_NTC_ ≠ 0. For viruses, a positive mNGS result was considered when the virus was not detected in NTC and at least one specific read was mapped to species or when RPM_sample_/RPM_NTC_ was >5 if the RPM_NTC_ ≠ 0.

In addition, positive mNGS results were further defined according to whether the detected microbes by mNGS were the most commonly reported pathogens or whether the infections caused by the microbes were in accordance with the clinical features of children. Guided by mNGS results, we also adjusted the therapeutic regimens, which were approved by the Ethical Review Committee.

### Diagnostic assessment

The definition of causative pathogens and final comprehensive clinical diagnoses were performed and confirmed by two to three clinical adjudicators independently, according to mNGS results, complete laboratory examinations, the treatment response of the patients, and clinical experiences. Based on the final comprehensive clinical diagnoses, the enrolled children were divided into the infectious diagnosis group, non-infectious diagnosis group, and indefinite clinical diagnosis group. The infectious diagnoses were based on 1) at least one of the results of culture, PCR, or antibody detection suggested pulmonary infections; or 2) for the patients without any laboratory examinations or with negative laboratory examinations, pneumonia was relieved after treatment according to the mNGS results. The non-infectious diagnoses were based on the following: 1) no pathogens were detected by both laboratory examinations and mNGS, and 2) the inflammation was relieved after the use of glucocorticoids or immunosuppressants. The indefinite clinical diagnosis group included patients whose clinical characteristics or laboratory examinations were not adequate for diagnoses and patients lost during follow-up duration. The final diagnoses of *M. pneumoniae* infection were also adjudicated according to the above pipeline. Taking final comprehensive clinical diagnoses as the reference standard, sensitivity, specificity, positive predictive value (PPV), negative predictive value (NPV), and total coincidence rate (TCR) of mNGS were calculated.

### Statistical analysis

Counts and percentages were presented for independent binomial variables. Mean ± standard error (SE) was calculated for continuous variables with normal distributions. *t*-Test was performed to evaluate categorical variables, and a *p*-value of <0.05 was considered statistically significant. The 95% confidence interval (CI) was calculated according to the formula:95% CI=Average±1.96SE. The data were analyzed using IBM SPSS 25.0 and R 4.1.1.

### Data availability

Sequencing data were deposited to the National Genomics Data Center under accession number CRA009297. The authors declare that the main data supporting the findings are available within this article. The other data generated and analyzed for this study are available from the corresponding authors upon reasonable request.

## Results

### Patient baseline

**Figure 1:**
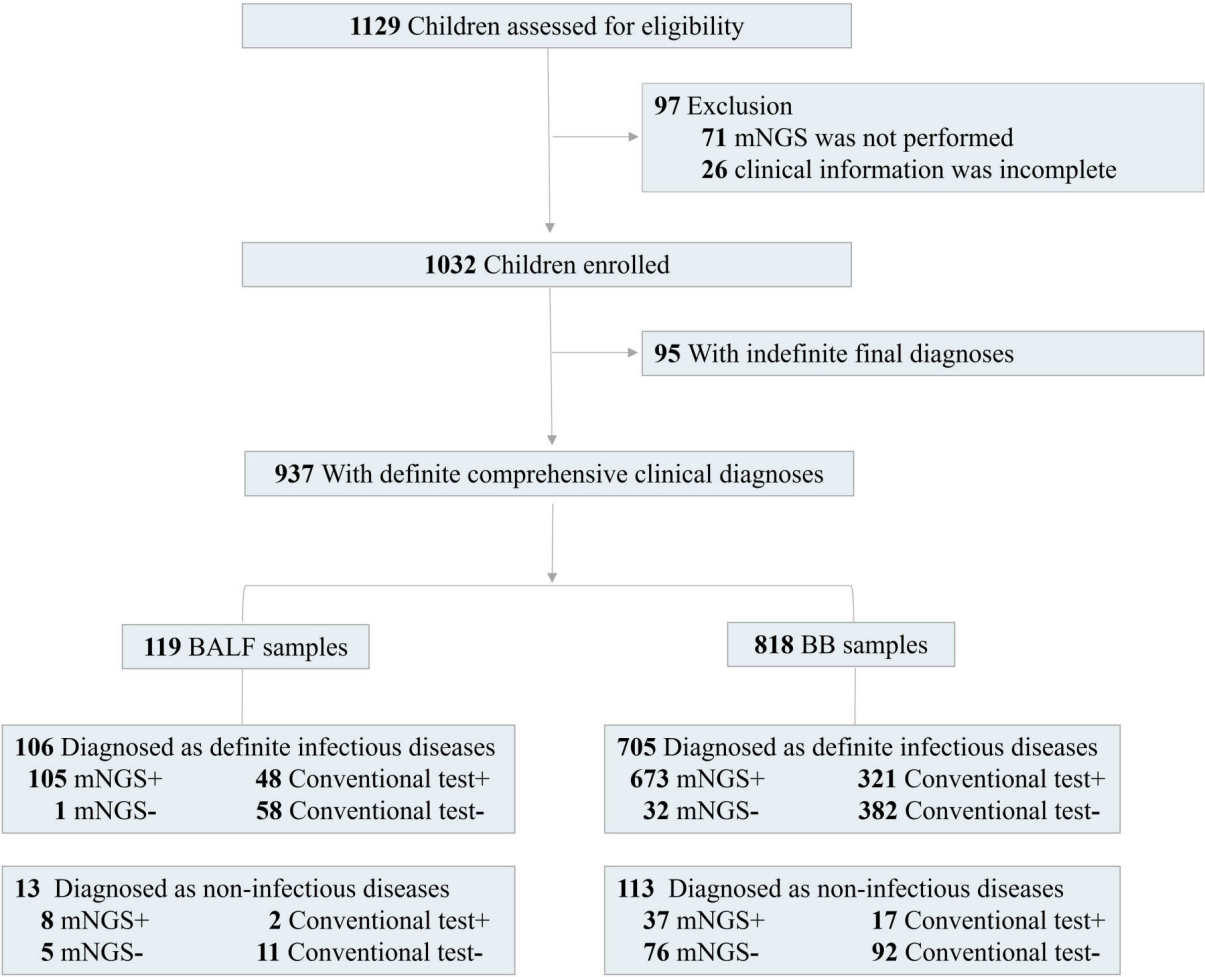
Flow diagram.

A total of 1,129 consecutive patients diagnosed with pneumonia from January 1, 2020, to April 30, 2022, were assessed for eligibility, while combined with exclusion criteria for samples, 1,032 children were enrolled for this study (**Figure 1**). There were 585 boys and 447 girls, and the average age was 5.2 ± 1.8 years (from 1 month to 16 years old). The average length of hospital stay was 16.1 ± 3.8 days (from 3 to 180 days). There were 905 (87.7%) children with severe pneumonia. A total of 648 children (62.8%) experienced a history of neonatal intensive care unit within 6 months (**Table 1**). A total of 304 patients (29.5%) experienced underlying diseases, including tracheomalacia/tracheostenosis (*n* = 133), congenital heart disease (*n* = 87), immunocompromised disease (*n* = 16), cranial nerve dysplasia (*n* = 11), inherited metabolic disease (*n* = 6), and allergic asthma/premature birth (*n* = 51).

**Table 1:** Clinical characteristics of enrolled patients.

Clinical characteristic	Count (%)/median ± SD
Sex (male)	585 (56.6%)
Age (years)	5.2 ± 1.8
Hospital stay (day)	16.1 ± 3.8
Severe pneumonia	905 (87.7%)
Cough	896 (86.8%)
Expectoration	207 (20.1%)
Dyspnea	725 (70.3%)
Fever	912 (88.4%)
History of neonatal intensive care unit	648 (62.8%)
WBC (×10^9^/L)	16.5 ± 3.8
Neutrophil granulocyte (×10^9^/L)	60.6 ± 18.5
CRP (mg/L)	23.1 ± 5.6
PCT (μg/L)	1.6 ± 0.4
ESR (mm/h)	33.4 ± 8.4
Congenital heart disease	87 (8.4%)
Tracheomalacia/tracheostenosis	133 (12.9%)
Immunocompromised diseases	16 (1.6%)
Cranial nerve dysplasia	11 (1.1%)
Inherited metabolic disease	6 (0.6%)
Allergic asthma/premature birth	51 (4.9%)

WBC, white blood cell; CRP, C-reactive protein; PCT, procalcitonin; ESR, erythrocyte sedimentation rate.

### Performance comparison between BALF mNGS and BB mNGS

**Figure 2:**
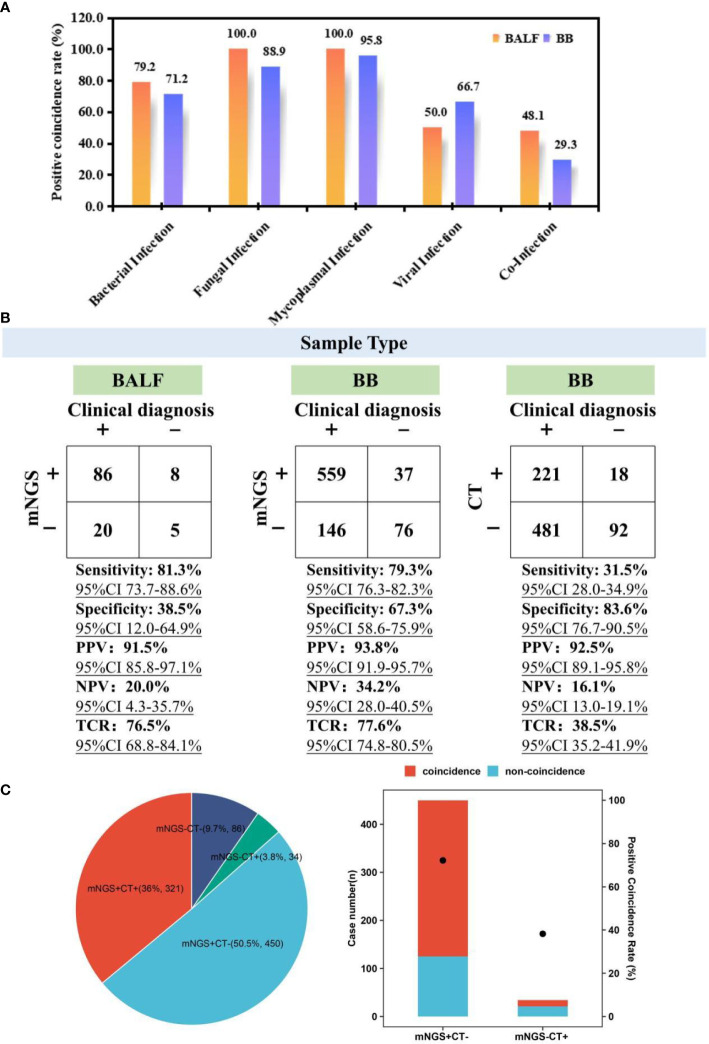
Performance comparison among BALF mNGS, BB mNGS, and CTs. **(A)** Positive coincidence rates of BALF mNGS and BB mNGS in different kinds of infections. **(B)** Performance of BALF mNGS, BB mNGS, and CTs against final clinical diagnoses. **(C)** Summary of the number of cases with positive or negative results revealed by mNGS and CTs. “+” and “−” represent positive and negative results, respectively. BALF, bronchoalveolar lavage fluid; mNGS, metagenomics next-generation sequencing; BB, bronchial brushing; CTs, conventional tests.

To evaluate the performance of mNGS, 119 children with BALF mNGS and 818 children with BB mNGS were included. According to the difference in the kinds of causative pathogens, infected children were further divided into bacterial infection (24/119 and 104/818), fungal infection (2/119 and 9/818), mycoplasma infection (50/119 and 453/818), viral infection (2/119 and 6/818), and co-infection (27/119 and 133/818) groups. For bacterial infection (**Figure 2A**), the positive coincidence rate of BALF mNGS (79.2%) against the final clinical diagnosis was slightly higher than that of BB mNGS (71.2%). A similar trend was also found in fungal (100.0% *vs*. 88.9%) and mycoplasma (100.0% *vs*. 95.8%) infections. In addition, BB mNGS (66.7%) exhibited better performance in diagnosing viral infection than BALF mNGS (50.0%), while BALF mNGS (48.1%) might be more suitable for diagnosing co-infection than BB mNGS (29.3%). The above results indicate that both BALF and BB samples are ideal samples for mNGS in diagnosing definite pulmonary infections.

Given the difference in the sampling process between BALF and BB samples, the overall performance of mNGS in diagnosing suspected pulmonary infections was further summarized (**Figure 2B**). Taking final comprehensive clinical diagnoses as reference standard, although the sensitivity of BALF mNGS [81.3% (95% CI 73.7-88.6%)] was slightly higher than that of BB mNGS [79.3% (95% CI 76.3%-82.3%)], specificity of BB mNGS [67.3% (95% CI 58.6%–75.9%)] was significantly higher than that of BALF mNGS [38.5% (95% CI 12.0%–64.9%)]. In addition, higher PPV [93.8% (95% CI 91.9%–95.7%)] and NPV [34.2% (95% CI 28.0%–40.5%)] were obtained by BB mNGS than BALF mNGS (91.5% (95% CI 85.8%–97.1%) and 20.0% (95% CI 4.3%–35.7%)). Most importantly, the TCR of BB mNGS [77.6% (95% CI 74.8%–80.5%)] was slightly higher than that of BALF mNGS [76.5% (95% CI 68.8%–84.1%)]. The above results further unravel that BB mNGS could be considered the preferred examination in diagnosing pulmonary infections in children.

### Advantage of BB mNGS over CTs

For the enrolled children, BB mNGS and CTs were simultaneously performed on 891 children (**Figure 2C**). There were only 321 children (36.0%) with both positive mNGS and CT results, while the number of children with both mNGS and CTs negative accounted for 9.7% (*n* = 86)In addition, mNGS can successfully detect microbes from more than half of the children (50.5%, *n* = 450) of whom the detection results by CTs were negative, while there were only 34 children (3.8%) with negative mNGS and positive CTs. Furthermore, a positive coincidence rate of mNGS in the children with positive mNGS and negative CTs against final clinical diagnoses can reach up to 72.2%, which was significantly higher than that of CTs (38.2%) in the children with negative mNGS and positive CTs. Further analysis also found that compared with those of BB mNGS, the sensitivity [31.5% (95% CI 28.0%–34.9%)], PPV [92.5% (95% CI 89.1%–95.8%)], NPV [16.1% (95% CI 13.0%–19.1%)], and TCR [38.5% (95% CI 35.2%–41.9%)] of CTs were lower.

### Diagnostic value of mNGS in *M. pneumoniae* infection

**Figure 3:**
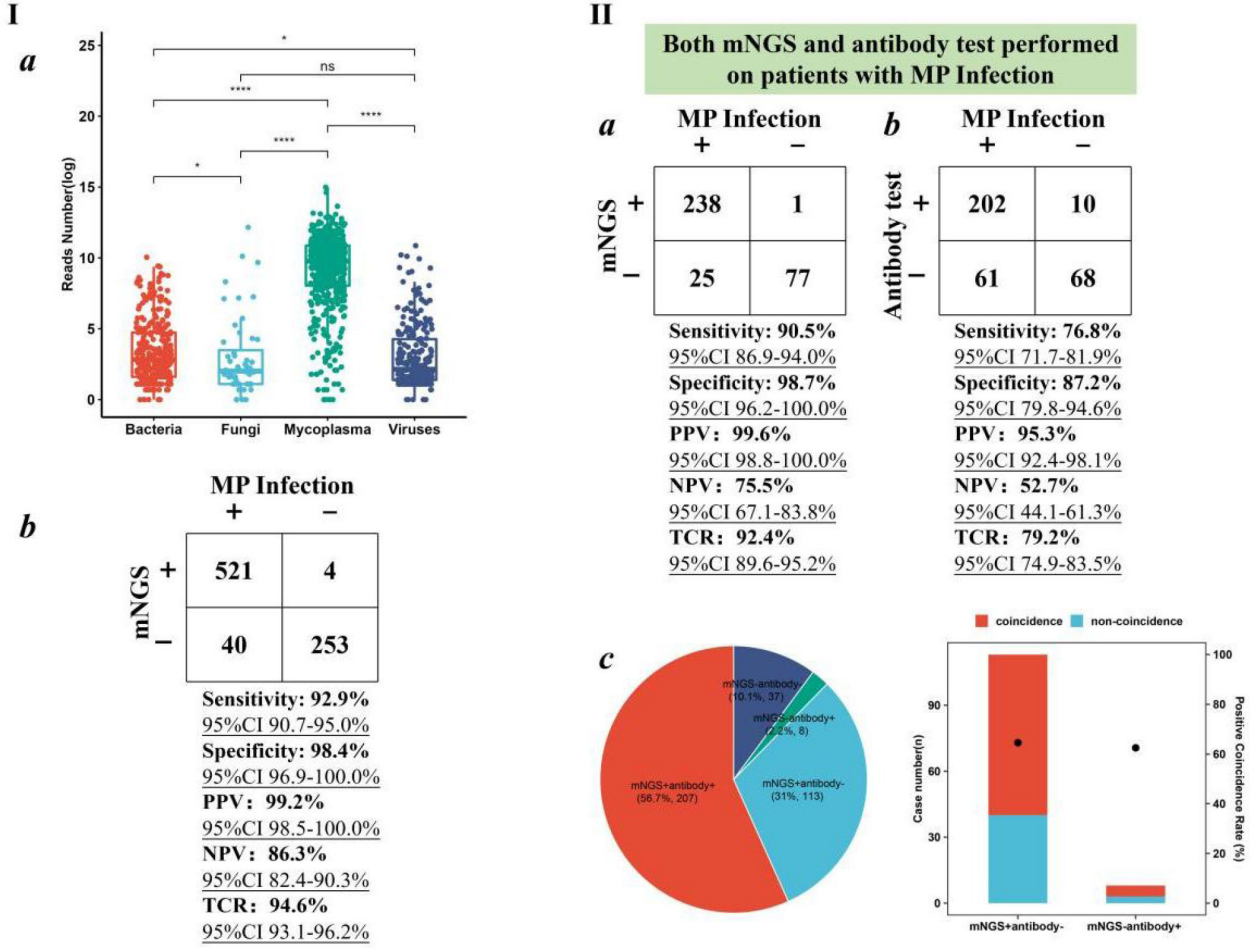
Performance of BB mNGS in diagnosing MP infection. **(I)** mNGS performance in the enrolled children with MP infection. **(II)** Performance of BB mNGS and antibody test against final clinical diagnoses. BB, bronchial brushing; mNGS, metagenomics next-generation sequencing; MP, *Mycoplasma pneumoniae*.

Given the epidemics of *M. pneumoniae*, the performance of BB mNGS and antibody test in diagnosing *M. pneumoniae* (MP) infection was further summarized (**Figure 3**). Among the 818 children with definite clinical diagnoses, 561 children were diagnosed with MP infection (**Figure 3Ib**). Taking final clinical diagnoses as reference standard, high sensitivity [92.9% (95% CI 90.7%–95.0%)], specificity [98.4% (95% CI 96.9%–100.0%)], PPV [99.2% (95% CI 98.5%–100.0%)], and NPV [86.3% (95% CI 82.4%–90.3%)] of BB mNGS were found, and the TCR even reached up to 94.6% (95% CI 93.1%–96.2%)]. The above results indicate that BB mNGS might be directly used for diagnosing or ruling out MP infection.

BB mNGS exhibited better performance than antibody test in diagnosing MP infection. Both BB mNGS and antibody test were performed on 341 children (**Figure 3II**). Sensitivity [90.5% (95% CI 86.9%–94.0%)], specificity [98.7% (95% CI 96.2%–100.0%)], PPV [99.6% (95% CI 98.8%–100.0%)], NPV [75.5% (95% CI 67.1%–83.8%)], and TCR [92.4% (95% CI 89.6%–95.2%)] of BB mNGS were found to be significantly higher than those of antibody test (76.8% (95% CI 71.7%–81.9%), 87.2% (95% CI 79.8%–94.6%), 95.3% (95% CI 92.4%–98.1%), 52.7% (95% CI 44.1%–61.3%)), and 79.2% (95% CI 74.9%–83.5%), respectively). High PPV of both BB mNGS and antibody test prompted us to identify in-depth reasons from the perspectives of the case.

Accordingly, we further analyzed the positive and negative results revealed by BB mNGS and antibody test. BB mNGS and antibody test can simultaneously detect *M. pneumoniae* from more than half of children (56.7%, *n* = 207), while only 37 children (10.1%) cannot be diagnosed by both BB mNGS and antibody test. In addition, the number of children with positive BB mNGS and negative antibody test (31.0%, *n* = 113) was more than that with positive antibody test and negative BB mNGS (2.2%, *n* = 8). Furthermore, the positive coincidence rate of BB mNGS in the children with positive BB mNGS and negative antibody test against final clinical diagnoses was 64.6%, which was slightly higher than that of antibody test (62.5%) in the children with negative BB mNGS and positive antibody test. Based on our findings, extensive application of BB mNGS in diagnosing pulmonary infections in children, especially MP infection, could be expected.

### Pathogen profiles

**Figure 4:**
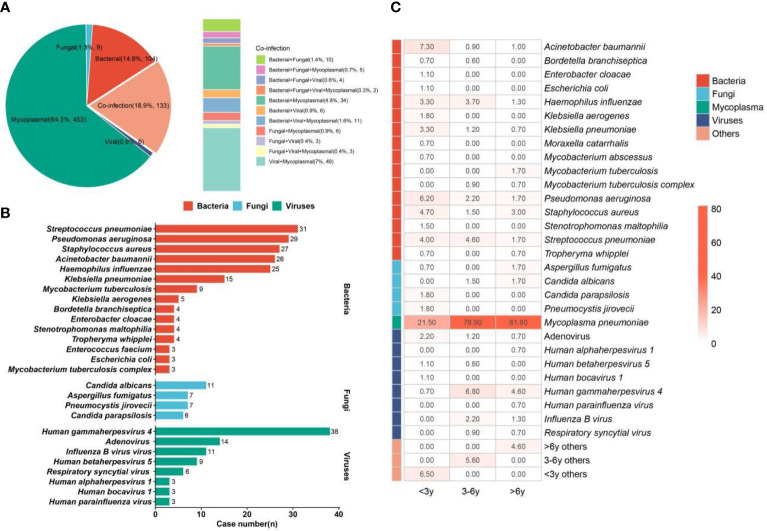
Infection type and pathogen profiles. **(A)** Different kinds of infections. **(B)** Bacteria, fungi, and viruses detected in the enrolled children. **(C)** Pathogen profiles in <3-year, 3–6-year, and >6-year groups. The number in the box represents the detection rate of some pathogens in the group.

During the epidemics of *M. pneumoniae*, more than 64% of pulmonary infections in children (*n* = 453) were caused only by *M. pneumoniae* (**Figure 4A**). Bacterial infection and co-infection accounted for 14.8% (*n* = 104) and 18.9% (*n* = 133), respectively. In addition, 1.3% (*n* = 9) and 0.9% (*n* = 6) of pulmonary infections were caused only by fungi and viruses, respectively. Among the co-infection group, most of the children were diagnosed with bacterial and mycoplasma co-infection (4.8%, *n* = 34) or viral and mycoplasma co-infection (7%, *n* = 49), and *M. pneumoniae* can be detected from 15.7% of children. The above results indicate that we should also pay attention to bacterial and viral infections during the epidemics of *M. pneumoniae*.

We summarized the detailed pathogen profiles during the epidemics of *M. pneumoniae* (**Figure 4B**). Among the children (*n* = 705) with definite clinical diagnoses, *M. pneumoniae* was diagnosed as a causative pathogen in 562 children. In addition, the most common bacterium was *Streptococcus pneumoniae* (*n* = 31), followed by *Pseudomonas aeruginosa* (*n* = 29), *Staphylococcus aureus* (*n* = 27), *Acinetobacter baumannii* (*n* = 26), and *Haemophilus influenzae* (*n* = 25). *Candida albicans* (*n* = 11) was the main fungal pathogen. For viruses, human gammaherpesvirus 4 (Epstein–Barr virus (EBV)) was detected in 38 children, while pulmonary infections in 14 and 11 children were infected by adenovirus and influenza B virus, respectively.

Given the heterogeneity of pathogen profiles caused by age, the children were further divided into the <3-year (*n* = 275), 3–6-year (*n* = 323), and >6-year (*n* = 302) groups, and the detection rates of different pathogens in different groups were also summarized (**Figure 4C**). The detection rate of *M. pneumoniae* in the >6-year group (81.8%) was higher than that in the 3–6-year (78.9%) and <3-year groups (21.5%). The highest detection rates of bacteria, fungi, and viruses were found in the <3-year, >6-year, and 3–6-year groups, respectively. For bacteria, *S. pneumoniae* and *H. influenzae* were mainly detected in the <3-year (4.0% and 3.3%) and 3–6-year (4.6% and 3.7%) groups, while *S. aureus* was mainly detected in the <3-year (4.7%) and >6-year (3.0%) groups. The highest detection rates of *A. baumannii* (7.3%), *P. aeruginosa* (6.2%), and *Klebsiella pneumoniae* (3.3%) were found in the <3-year group. For fungi, *Candida parapsilosis* (1.8%) and *Pneumocystis jirovecii* (1.8%) were only detected in the <3-year group, while *C. albicans* cannot be found in this group. In addition, the highest detection rate of EBV (6.8%) was found in the 3–6-year group, which can also cause infection in 4.6% of children aged >6 years.

### The best timing for mNGS tests

**Figure 5:**
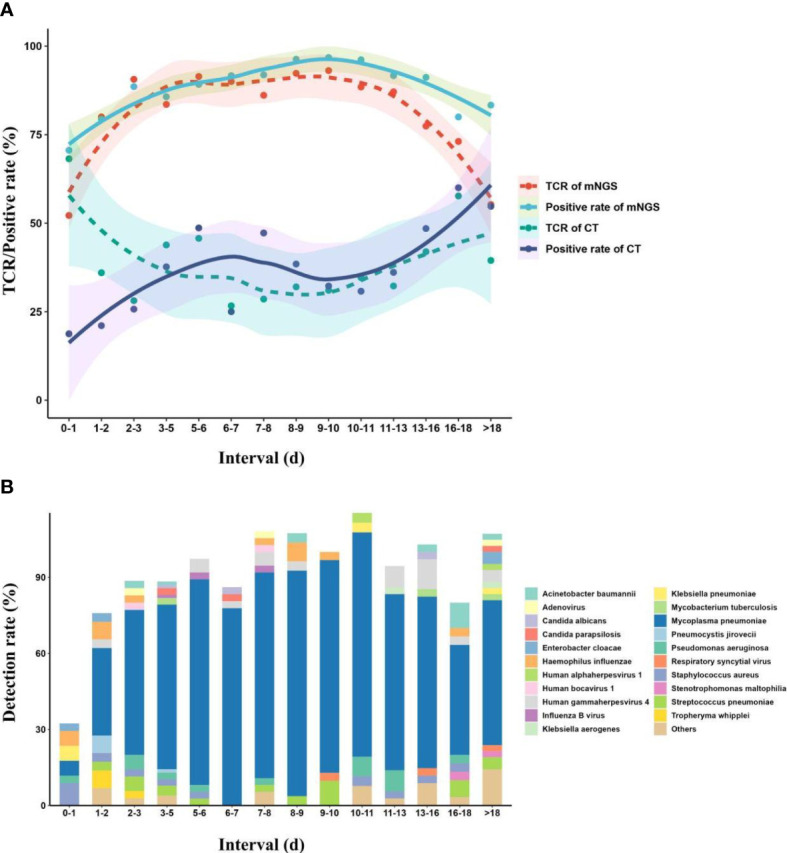
Effect of continuous empirical therapy on mNGS detection and pathogen succession. **(A)** Changes in mNGS performance with the duration of empirical therapy before mNGS tests. **(B)** Pathogen succession with the use of empirical therapy. mNGS, metagenomics next-generation sequencing.

Almost all of the children enrolled in this study were treated with empirical therapy before mNGS tests, and the children were divided into different interval groups according to the duration of empirical therapy before mNGS tests. The TCR of mNGS, positive rate of mNGS, TCR of CTs, and positive rate of CTs were fitted over the duration using local polynomial regression fitting (Cleveland et al., 1992) (**Figure 5**). The fitting model shows that with the increase in duration, the TCR of mNGS increased and can reach up to 91.4% at the duration of 5–6 days, which can still maintain a stable level of approximately 90% until the duration of 11–13 days. When the duration was more than 13 days, the TCR of mNGS significantly decreased, while the highest TCR of CTs (68.2%) was found at the duration of 0–1 day. In addition, with the increase in the duration, the diversity of pathogens showed an opposite trend as the TCR of mNGS, and only four kinds of pathogens were found at the duration of 6–7 days. According to the above findings, we propose that the best timing for mNGS detection in pulmonary infections in children ranged from 5 to 7 days after the start of continuous anti-microbial therapy.

**Figure 6:**
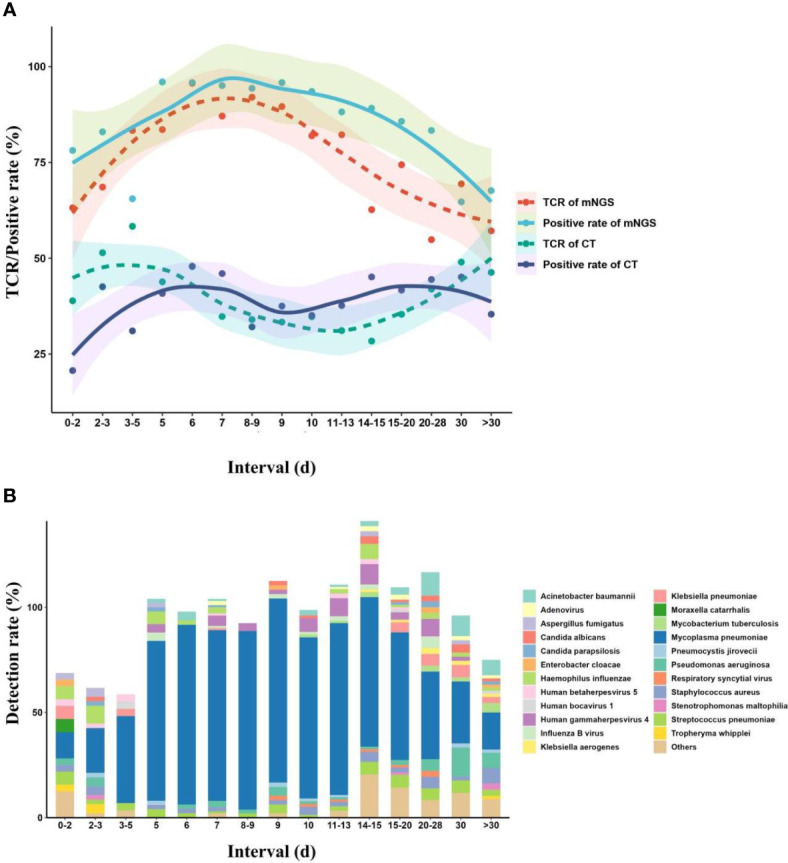
Effects of onset duration on mNGS detection and pathogen succession. **(A)** Changes in mNGS performance with the duration of onset before mNGS test. **(B)** Pathogen succession with the onset duration. mNGS, metagenomics next-generation sequencing.

Meanwhile, we also divided the children into different interval groups according to the duration from onset to mNGS tests (**Figure 6**). The fitting model showed that with the increase of duration, the TCR of mNGS (95.7%) peaked at the duration of 6 days, and a TCR of 92% was found at the duration of 8–9 days, while the highest TCR of CTs (58.3%) was found at the duration of 3–5 days, which can maintain at a stable level of approximately 50% at the duration of 0–6 days. From the duration of 7 days, the TCR of CTs decreased. In addition, with the increase in the duration, the diversity of pathogens also showed an opposite trend as the TCR of mNGS, and we also found only four kinds of pathogens at the duration of 8–9 days. Accordingly, we propose that the mNGS test should be performed at a duration of 6–9 days from onset to the mNGS test.

### Changes in treatment strategies

**Figure 7:**
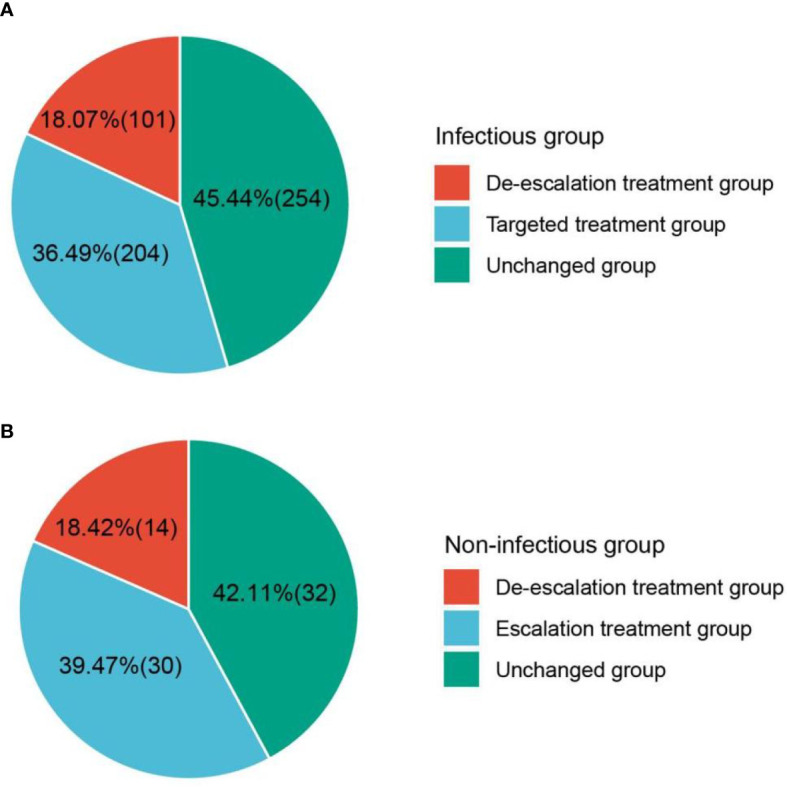
Effect of mNGS on treatment. **(A)** Beneficial impact of mNGS on most children in infectious group. **(B)** Clinical benefit of mNGS on the children in non-infectious group. mNGS, metagenomics next-generation sequencing.

mNGS plays an important role in providing a reference for clinical therapy. The effects of mNGS on both infectious and non-infectious groups were summarized (**Figure 7**). mNGS exhibited a beneficial impact on most of the children in the infectious group, including 18.1% of children (*n* = 101) with de-escalation treatment and 36.5% of children (*n* = 204) with targeted treatment. In addition, the empirical treatment on 45.4% of children (*n* = 254) was not changed. However, there was no significant clinical benefit of mNGS on the children in the non-infectious group. Although both mNGS and CTs did not detect any pathogens, escalation treatment was performed on 39.5% of children (*n* = 30), and the treatment on 42.1% of children (*n* = 32) was not adjusted. De-scalation treatment was only performed on 18.4% of children (*n* = 14). Given the good prognoses of enrolled children (clinical symptoms of 888 out of 900 children significantly improved), we propose that mNGS should be extensively applied in diagnosing pulmonary infections in children.

## Discussion

To the best of our knowledge, this is the first report to evaluate the performance of BB mNGS in diagnosing pulmonary infections in children on a large scale. Compared with those of BALF mNGS, higher specificity (67.3% (95% CI 58.6%–75.9%)) and TCR (77.6% (95% CI 74.8%–80.5%)) of BB mNGS unravel that BB mNGS could be considered the preferred examination in diagnosing pulmonary infections in children, especially for diagnosing or ruling out MP infection. During the epidemics of *M. pneumoniae*, the detection rate of *M. pneumoniae* in the >6-year group (81.8%) was higher than that in the 3–6-year (78.9%) and <3-year groups (21.5%). The highest detection rates of bacteria, fungi, and viruses were found in the <3-year, >6-year, and 3–6-year groups, respectively. The fitting model shows that the mNGS detection in pulmonary infections in children should be performed at the duration of 5–7 days after the start of continuous anti-microbial therapy or at the duration of 6–9 days from onset to the mNGS test.

Our results confirmed that BB mNGS was superior to BALF mNGS and CTs in diagnosing pulmonary infections in children, especially for the detection of *M. pneumoniae*. Low detection efficiency in pathogen culture (Ge et al., 2021), limited number of pathogens detected in a single experiment using hypothesis-based PCR (Marcilla-Vazquez et al., 2018), and possible false-negative results at an early stage by antibody methods (Ge et al., 2021) hindered the accurate and timely detection of pathogens using CTs. Tang et al. found that the positive rate of MP-IgM was only 40% in detecting MP infection in children (Tang et al., 2021). In addition, the detection performance of CTs, especially the culture, was sensitive to anti-infective therapy, whose effect on mNGS detection was hysteretic (Zhang et al., 2020; Ge et al., 2021). Zhang et al. (Zhang et al., 2020) also proposed that the detection of mNGS was affected after anti-microbial treatment of >4 days on adults with central nervous system (CNS) infection. Furthermore, the BB sampling process can realize relatively accurate microbial collection from pulmonary focal sites (Wang et al., 2020), while some colonizing microbes derived from the lung, respiratory tract, or oral cavity are included during the BALF sampling process. Chen et al. found that the sensitivity of BALF mNGS can reach up to 88.9%, while the specificity was only 14.9% (Chen et al., 2021b), which was consistent with our results. Accordingly, the use of BB mNGS can ensure the reliability and accuracy of our data, and we propose that BB mNGS could be considered the preferred examination in diagnosing pulmonary infections in children.

We found that during the epidemics of *M. pneumoniae*, the detection rate of *M. pneumoniae* increased in children with age, which was consistent with previous studies (Alexander et al., 1966; Foy et al., 1966; Basarab et al., 2014; Jain et al., 2015; Shim, 2020; Tang et al., 2021). Tang et al. found that there were few significant MP infections in children aged <3 years, while school-aged children and adolescents were the most vulnerable to MP infection (Tang et al., 2021). Shim et al. also pointed out that the incidence of MP pneumonia peaked in school-aged children (Shim, 2020). It was reported that MP pneumonia accounts for 7%–20% of community-acquired pneumonia cases in children aged 3–15 years (Don et al., 2010; Shim, 2020). Jain et al. also found that the incidence rate of MP pneumonia in children aged 5 or older can reach up to 19%, which was much higher than that (3%) in children younger than 5 years (Jain et al., 2015). We found that during the epidemics of *M. pneumoniae*, the highest detection rate (81.8%) of *M. pneumoniae* was found in the >6-year group, followed by the 3–6-year (78.9%) and <3-year groups (21.5%). The above findings might be attributed to the host’s immune status, bacterial colonization in the host, and exposure to bacteria-contaminated settings (Shim, 2020).

We should also pay attention to bacterial and viral infections during the epidemics of *M. pneumoniae*. A previous study found that viruses and bacteria were detected in 81% and 63%, respectively, of children with lower respiratory tract infections, and 52% of children had bacterial and viral co-infection (Tsitsiklis et al., 2022). In addition, Jain et al. also found that viruses (66%) were more commonly detected than bacteria (8%) in children with community-acquired pneumonia (Jain et al., 2015). We found that bacterial and viral infections only accounted for 14.8% and 0.9%, respectively, of pulmonary infections, and the highest detection rates of bacteria, fungi, and viruses were found in the <3-year, >6-year, and 3–6-year groups, respectively. At the genus level, the highest detection rates of *A. baumannii*, *P. aeruginosa*, and *K. pneumoniae* found in the <3-year group unravel that hospital-acquired infection is one of the causes of pneumonia in those children (Mamishi et al., 2019), reminding us to pay attention to hospital-acquired pneumonia. Respiratory syncytial virus is the most common virus detected in children with pneumonia (Pneumonia Etiology Research for Child Health (PERCH) Study Group, (2019); Jain et al., 2015; Tsitsiklis et al., 2022), which was contrary to our result. The highest detection of virus was EBV during the epidemics of *M. pneumoniae* in our study, while a retrospective study found that co-infection of EBV and *M. pneumoniae* posed a higher risk for prolonged symptoms (Xu et al., 2020). Given the complexity of infections in children, unbiased mNGS should be extensively used for accurate and timely diagnoses to improve the prognoses of children.

We propose that mNGS detection in pulmonary infections in children should be performed at the duration of 5–7 days after the start of continuous anti-microbial therapy or at the duration of 6–9 days from onset to mNGS test. Chen et al. also found that the best timing for mNGS detection in neonatal infections ranged from 1 to 3 days, rather than 0 days, after the start of continuous anti-microbial therapy (Chen et al., 2022). Given the inevitability and indispensability of empirical therapy, most children received empirical therapy after the onset of infection (Nascimento-Carvalho and Nascimento-Carvalho, 2019). The initial empirical therapy, which inhibits the cell wall synthesis of some pathogens or directly kills part of pathogens (Tomasz and Waks, 1975), can significantly influence the detection of CTs, while its effect on mNGS detection was hysteretic (Zhang et al., 2020; Ge et al., 2021). In addition, a low pathogen load at the onset of infection may make the mNGS unable to detect some trace pathogens. The above may be the reasons why the TCR of mNGS increased and the TCR of CTs decreased, with the increase in duration from both the start of continuous anti-microbial therapy and the onset of the mNGS test.

However, long-time and continuous anti-microbial therapy may result in the generation of drug-resistant pathogens, hospital-acquired infections (Kalil et al., 2016), and secondary infections with some trace pathogens (Fattorini et al., 2020). Many microbes can be separated or detected from children in that period using CTs (Fattorini et al., 2020), while pathogen succession *in vivo* might make it difficult to accurately define causative pathogens from the detected microbes by mNGS according to complex clinical symptoms, or some causative pathogens with low (secondary infection) or zero (disappeared) load cannot be detected by mNGS. The increase in the TCR of CTs and the decrease in the TCR of mNGS may be attributed to the above reasons. In addition, Zhang et al. (Zhang et al., 2020) also proposed that anti-microbial treatment of >4 days on adults with CNS infection significantly affected the detection of mNGS. The dynamic process suggested that mNGS should be performed at the right timing to decrease the therapy time and improve the prognoses of children.

## Conclusions

Our study emphasized that BB mNGS had overall superiority to CTs in causative pathogen detection from children with pulmonary infections. Our findings unravel that BB mNGS could be considered the preferred examination in diagnosing pulmonary infections in children, especially for diagnosing or ruling out MP infection. In addition, we also propose that the mNGS detection in pulmonary infections in children should be performed at the duration of 5–7 days after the start of continuous anti-microbial therapy or at the duration of 6–9 days from onset to the mNGS test. Based on our findings, extensive application of BB mNGS in diagnosing pulmonary infections in children could be expected.

## Data availability statement

The datasets presented in this study can be found in online repositories. The names of the repository/repositories and accession number(s) can be found in the article/supplementary material.

## Ethics statement

This study was reviewed and approved by the Ethical Review Committee of Children’s Hospital Affiliated to Shandong University (approval no. SDFE-IRB/P-2022017). Written informed consent to participate in this study was provided by the participants’ legal guardian/next of kin.

## Author contributions

SW designed the paper. CZ, ZL, MW, and JZ drafted the manuscript. CZ, ZL, MW, and WY carried out the clinical care and management of the patients. JZ, BH, and HL performed the mNGS tests and analyzed the data. JZ and SW revised the manuscript. All authors contributed to the article and approved the submitted version.
